# Charge of Murder—Knife Found in the Abdomen of a Female

**Published:** 1829-07-01

**Authors:** 


### XXIII.

### Charge of Murder—Knife found in the Abdomen of a Female.

Forensic medicine has flourished of late, and some most extraordinary medical evidence has been given. The alleged murder of Mr. Neale by Butler the soldier, had hardly been disposed of, when a most mysterious trial was reported from Bury St. Edmunds, some particulars of which are worthy of record in a medical journal.

On the evening of the 11th of October last, (Sunday,) a butler and a female servant quarrelled in the pantry, and the former having a small tray, containing four knives and forks, in his hand, threw them down at the feet (as he alleged) of the female, in order to frighten her. The latter went out of the pantry for about three minutes, and then returned, saying she was wounded. The butler inquired where—and she replied in her side. She borrowed a silk handkerchief from the butler, and then went into the kitchen where there were prayers. In the night she was found to be ill, and next day a surgeon, Mr. Bartlet, was called to the patient. He found a wound of an inch in length, between the spine of the ilium and the margin of the ribs, whence there had been considerable hæmorrhage. Mr. B. dressed the wound, without examining its depth or direction with a probe. She had been sick, and complained of great pain in the abdomen. She died at half-past one o'clock on Wednesday morning. On examination, Mr. Bartlett "found a knife lying transversely in the body, with the point a little turned upwards. It had penetrated the stomach, one of the small, and one of the large intestines. It had produced very extensive inflammation, which occasioned her death." It appeared that the clothes, including even the stays, were perforated by the knife. It also appeared that the female in question was of a most passionate temper—that she had a great aversion to the butler's wife—and accused the man that day of being to see his wife, to whom she applied a most opprobrious epithet. These circumstances induce us to conclude that the deceased committed suicide under some mental hallucination or momentary gust of passion—perhaps of jealousy. It is quite impossible to suppose that the butler could have perpetrated such a crime, and worked a common cheese-knife through the clothes of a female, so as to be completely buried in the abdomen of his victim—and all this against her consent! The idea is preposterous. Nothing but a fit of desperate insanity could have enabled the deceased herself to accomplish such a horrible and destructive operation. Neither do we attach much importance to her dying declaration that the knife found its way into the position above described merely by being thrown at her from a tray. She absolved the butler from all design upon her life, and freely forgave him on her death-bed. The case has no parallel, except in the annals of insanity—and under that head we would certainly range the transaction, though it does not appear to have been so viewed by either judge, jury, or medical witnesses. The man was acquitted.

